# Supplementary material for: Prognostic Significance of p16 and Its Relationship with Human Papillomavirus Status in Patients with Penile Squamous Cell Carcinoma: Results of 5 Years Follow-Up
Source: Cancers (Basel). 2022 Dec 7;14(24):6024. doi: 10.3390/cancers14246024 (PMC9775956; doi:10.3390/cancers14246024)
Supplement: Supplementary file 1 [file cancers-14-06024-s001.zip › cancers-2027229-supplementary.pdf]

cancers

MDPI

Table S1. Patient Characteristics.

Patient Characteristics		
**Covariate**	**Status**	**N (%)**
HPV	negative	96(67.1%)
	positive	47(32.9%)
p16	negative	98(68.5%)
	positive	45(31.5%)
Ethnicity	Caucasian	88(61.5%)
	African American	14(9.8%)
	Hispanic	40(28%)
	Asian	1(0.7%)
Tobacco	Never	56(39.2%)
	Active/Former	87(60.8%)
Phimosis	No	81(57.4%)
	Yes	60(42.6%)
Circumcised	No	101(71.6%)
	Yes	40(28.4%)
Clinical T stage (cT)	1	45(31.7%)
	2	78(54.9%)
	3	16(11.3%)
	4	3(2.1%)
Clinical N stage (cN)	0	74(51.7%)
	1	28(19.6%)
	2	12(8.4%)
	3	29(20.3%)
Pathological T stage (pT)	1	37(26.6%)
	2	71(51.1%)
	3	26(18.7%)
	4	5(3.6%)
Pathological N stage (pN)	0	71(51.1%)
	1	16(11.5%)
	2	12(8.6%)
	3	40(28.8%)
Histology	usual SCC	117(81.8%)
	basaloid	15(10.5%)
	pap. NOS	3(2.1%)
	sarcomatoid	1(0.7%)
	verrucous	5(3.5%)
	warty	2(1.4%)
Grade	1	27(18.9%)

	2	55(38.5%)
	3	61(42.7%)
LVI	No	71(50.7%)
	Yes	69(49.3%)
Neoadjuvant Chemotherapy	No	97(67.8%)
	Yes	46(32.2%)
Response to Chemotherapy	CR	4(8.7%)
	PR	16(34.8%)
	SD	10(21.7%)
	PD	15(32.6%)
Greater than 75% staining p16	No	98(68.5%)
	Yes	45(31.5%)
p16 IHC staining pattern	0	58(40.6%)
	1	36(25.2%)
	2	17(11.9%)
	3	32(22.4%)
Survival Status	No Evidence of Disease	60(42.6%)
	Died of Disease	42(29.8%)
	Died	28(19.9%)
	Alive with Disease	11(7.8%)

Table S2. Patient characteristics associated with HPV status. Some differences observed but percentages are similar to patient characteristics associated with p16 status. .

Covariate	Status	HPV = negative	HPV = positive	Fisher's Exact Test P-value (2-Tail)
p16	Negative	87(88.8%)	11(11.2%)	< 0.001
	Positive	9(20%)	36(80%)	
Age at diagnosis (median, min, max)		96 (58.3, 48.0, 68.6)	47 (59.7, 49.6, 68.5)	0.58
Number of lymph nodes collected (median, min, max)		80 (30.0, 20.0, 41.5)	37 (27, 20, 37)	0.54
Total Positive Nodes (median, min, max)		80 (1, 0, 2.5)	38 (1, 0, 2)	0.89
Ethnicity	Caucasian	54(61.4%)	34(38.6%)	0.050
	African American	8(57.1%)	6(42.9%)	
	Hispanic	33(82.5%)	7(17.5%)	
	Asian	1(100%)		
Tobacco Use	Never	42(75%)	14(25%)	0.144
	Active/Former	54(62.1%)	33(37.9%)	
Phimosis	No	49(60.5%)	32(39.5%)	0.075
	Yes	45(75%)	15(25%)	
Circumcised	No	71(70.3%)	30(29.7%)	0.168
	Yes	23(57.5%)	17(42.5%)	
cT stage	1	33(73.3%)	12(26.7%)	0.242
	2	52(66.7%)	26(33.3%)	
	3	8(50%)	8(50%)	
	4	3(100%)		
cN stage	0	48(64.9%)	26(35.1%)	0.955
	1	20(71.4%)	8(28.6%)	
	2	8(66.7%)	4(33.3%)	
	3	20(69%)	9(31%)	
pT stage	1	29(78.4%)	8(21.6%)	0.382
	2	46(64.8%)	25(35.2%)	
	3	16(61.5%)	10(38.5%)	
	4	3(60%)	2(40%)	
pN stage	0	50(70.4%)	21(29.6%)	0.812
	1	12(75%)	4(25%)	
	2	7(58.3%)	5(41.7%)	
	3	27(67.5%)	13(32.5%)	
Grade	1	23(85.2%)	4(14.8%)	< 0.001
	2	43(78.2%)	12(21.8%)	
	3	30(49.2%)	31(50.8%)	
Histology	usual SCC	83(70.9%)	34(29.1%)	< 0.001

	basaloid	3(20%)	12(80%)	
	pap. NOS	3(100%)		
	sarcomatoid	1(100%)		
	verrucous	5(100%)		
	warty	1(50%)	1(50%)	
IHC 75% staining	Less than	87(88.8%)	11(11.2%)	< 0.001
	Greater than	9(20%)	36(80%)	
IHC Staining Pattern	0	53(91.4%)	5(8.6%)	< 0.001
	1	31(86.1%)	5(13.9%)	
	2	8(47.1%)	9(52.9%)	
	3	4(12.5%)	28(87.5%)	

Table S3. Patient characteristics associated with p16 status. Some differences observed but percentages are similar to patient characteristics associated with HPV status. .

Covariate	Status	p16 = negative	p16 = positive	Fisher's Exact Test P-value (2-Tail)
HPV	negative	87(90.6%)	9(9.4%)	< 0.001
	positive	11(23.4%)	36(76.6%)	
Age at diagnosis (median, min, max)		98 (59.5, 45.1, 68.8)	45 (58.2, 49.8, 68.3)	0.811
Number of lymph nodes collected (median, min, max)		81 (30.0, 20.0, 41.0)	36 (27.0, 18.0, 39.0)	0.580
Total Positive Nodes (median, min, max)		81 (1, 0, 3)	37 (1, 0, 2)	0.522
Ethnicity	Caucasian	57(64.8%)	31(35.2%)	0.050
	African American	7(50%)	7(50%)	
	Hispanic	33(82.5%)	7(17.5%)	
	Asian	1(100%)		
Tobacco Use	Never	44(78.6%)	12(21.4%)	0.044
	Active/Former	54(62.1%)	33(37.9%)	
Phimosis	No	48(59.3%)	33(40.7%)	0.011
	Yes	48(80%)	12(20%)	
Circumcised	No	72(71.3%)	29(28.7%)	0.231
	Yes	24(60%)	16(40%)	
cT stage	1	32(71.1%)	13(28.9%)	0.131
	2	55(70.5%)	23(29.5%)	
	3	7(43.8%)	9(56.3%)	
	4	3(100%)		
cN stage	0	50(67.6%)	24(32.4%)	0.946
	1	20(71.4%)	8(28.6%)	
	2	9(75%)	3(25%)	
	3	19(65.5%)	10(34.5%)	
pT stage	1	27(73%)	10(27%)	0.271
	2	47(66.2%)	24(33.8%)	
	3	15(57.7%)	11(42.3%)	
	4	5(100%)		
pN stage	0	49(69%)	22(31%)	0.721
	1	11(68.8%)	5(31.3%)	
	2	7(58.3%)	5(41.7%)	
	3	30(75%)	10(25%)	
Grade	1	23(85.2%)	4(14.8%)	0.002
	2	43(78.2%)	12(21.8%)	
	3	32(52.5%)	29(47.5%)	

Histology	usual SCC	87(74.4%)	30(25.6%)	< 0.001
	basaloid	1(6.7%)	14(93.3%)	
	pap. NOS	3(100%)		
	sarcomatoid	1(100%)		
	verrucous	5(100%)		
	warty	1(50%)	1(50%)	
IHC 75% staining	Less than	98(100%)		< 0.001
	Greater than		45(100%)	
IHC Staining Pattern	0	58(100%)		< 0.001
	1	36(100%)		
	2	4(23.5%)	13(76.5%)	
	3		32(100%)	

Table S4. Samples with discordance between HPV and p16 status. The columns are from right to left – sample ID, HPV status, p16 status determined by HS analysis, percentage of p16 staining, p16 staining pattern, and the HPV analysis used to determine HPV status.

Sample ID	HPV Status	p16 Status	% Staining	Staining pattern	Cobas or RNAscope
24	Negative	Positive	> 75%	2	Cobas
29	Negative	Positive	>75%	2	RNAscope
46	Negative	Positive	>75%	3	RNAscope
65	Negative	Positive	>75%	2	Cobas
93	Negative	Positive	>75%	2	Cobas
126	Negative	Positive	>75%	3	RNAscope
149	Negative	Positive	>75%	3	RNAscope
74	Negative	Positive	>75%	2	RNAscope
119	Negative	Positive	>75%	3	RNAscope
137	Positive	Negative	< 75%	2	RNAscope
140	Positive	Negative	< 75%	1	RNAscope
21	Positive	Negative	< 75%	1	Cobas
61	Positive	Negative	< 75%	0	Cobas
19	Positive	Negative	< 75%	1	Cobas
20	Positive	Negative	< 75%	1	Cobas
25	Positive	Negative	< 75%	0	Cobas
35	Positive	Negative	< 75%	1	Cobas
99	Positive	Negative	< 75%	0	Cobas
101	Positive	Negative	< 75%	0	Cobas
112	Positive	Negative	< 75%	0	Cobas

Table S5. Samples that were found to have a p16 staining pattern of 2. We have separated out the samples that p16 and HPV status matched (Sample ID 1 through 23) and samples that p16 and HPV did not match (Sample ID 24 through 137).

Sample Number	Sample ID	HPV Status	p16 Status	% Staining	Staining Pattern
1	1	Positive	Positive	>75%	2
2	30	Positive	Positive	>75%	2
3	58	Positive	Positive	>75%	2
4	68	Positive	Positive	>75%	2
5	88	Positive	Positive	>75%	2
6	105	Positive	Positive	>75%	2
7	111	Positive	Positive	>75%	2
8	148	Positive	Positive	>75%	2
9	143	Negative	Negative	< 75%	2
10	151	Negative	Negative	< 75%	2
11	23	Negative	Negative	< 75%	2
1	24	Negative	Positive	>75%	2
2	29	Negative	Positive	>75%	2
3	65	Negative	Positive	>75%	2
4	74	Negative	Positive	>75%	2
5	93	Negative	Positive	>75%	2
6	137	Positive	Negative	< 75%	2
